# *De Novo* Whole-Genome Sequence of Micromonospora carbonacea JXNU-1 with Broad-Spectrum Antimicrobial Activity, Isolated from Soil Samples

**DOI:** 10.1128/genomeA.00174-15

**Published:** 2015-04-09

**Authors:** Yun Jiang, Yun-hong Huang, Zhong-er Long

**Affiliations:** College of Life Science, Jiangxi Normal University, Nanchang, Jiangxi, Zhōngguó, China

## Abstract

Micromonospora carbonacea JXNU-1 is an actinomycete with broad-spectrum antimicrobial activity, isolated from soil samples from the farmland in the area of Yaohu Lake in Nanchang, China. Here, we report the whole-genome sequence of M. carbonacea JXNU-1.

## GENOME ANNOUNCEMENT

Micromonospora carbonacea JXNU-1 is an actinomycete with broad-spectrum antimicrobial activity. The strain was initially isolated from soil samples from farmland in the area of Yaohu Lake in Nanchang, China (1). The single antibiotic component from the strain showed broad-spectrum antimicrobial activity and featured nucleosides (2), unlike the other strain of M. carbonacea (3–7).

The genomic DNA from M. carbonacea JXNU-1 was obtained from mycelia cultured in liquid supplemented minimal medium (ISP_2_ medium) under shaken conditions at 200 rpm at 28°C for 5 days. The Illumina HiSeq 2500 and Illumina MiSeq platforms were used to construct 2 different genomic DNA libraries according to the manufacturer’s instructions. Long-insert (about 5-kb) libraries were sequenced by the paired-end mode using the Illumina HiSeq 2500, and the short-insert (500-bp) libraries were sequenced by the paired-end mode with Illumina MiSeq. DNA sequencing resulted in 3,487 Mb raw reads, of which 2,393 Mb reads passed stringent quality filters and were used to create the final assembly using the SOAPdenovo alignment tool (version 2.0) (8), with multiplex PCR used to close the gaps (9, 10). The assembly consists of 8 scaffolds, including 9 large contigs (sum, 7.63 Mb; *N*_50_, 1.11 Mb; maximum length, 2.31 Mb).

The whole genome of M. carbonacea JXNU-1 contains a single circular chromosome of 7,635,725 bp, with an average G+C content of 73.85%. A total of 6,444 coding sequences (CDs) were identified by GeneMarkS (11, 12). In addition, there are 27 interspersed repeats (IRs) with 2,731 bp and 4,100 tandem repeats (TRs) with 196,933 bp in the genome, respectively. We also used rRNAmmer (13), tRNAscan (14), and Rfam to identify noncoding RNAs; 6 rRNAs, 51 tRNAs, and 23 sRNAs were found in genome, respectively. In addition, 16 genomic islands (GIs) and 22 clustered regularly interspaced short palindromic repeats (CRISPR) were predicted using IslandPath-DIOMB software and CRISPRFinder (http://crispr.u-psud.fr/), respectively.

### Nucleotide sequence accession numbers.

This whole-genome shotgun project has been deposited at DDBJ/EMBL/GenBank under the accession number JXSX00000000. The version described in this paper is the first version, JXSX01000000.
